# Comparison of Albumin and Fresh Frozen Plasma as Colloid Therapy in Patients With Major Burns

**DOI:** 10.7759/cureus.33485

**Published:** 2023-01-07

**Authors:** Selahattin Vural, Cinar A Yasti, Mete Dolapçı

**Affiliations:** 1 General Surgery, Giresun University Faculty of Medicine, Giresun, TUR; 2 Department of General Surgery, University of Health Sciences, Ankara, TUR; 3 Department of General Surgery, Ufuk University, Ankara, TUR

**Keywords:** burn treatment, mortality, fresh frozen plasma (ffp), albumin, major burn

## Abstract

Background: Burn injuries are one of the main causes of morbidity and mortality throughout the world, and burn patients have higher chances of recovery if they are treated with effective fluid and colloid management. Fresh frozen plasma (FFP) and albumin suspension used as a colloid treatment agent are very useful for the treatment of severe burns.

Methods: This retrospective clinical trial was conducted at the Numune Education and Research Hospital Burn Center, Ankara, Turkey. Two hundred and nine patients who had severe burns that involved more than 30% of their total body surface area (TBSA) were studied. After the first 24 hours, 13 patients were deceased, leaving 196 patients remaining in the study, including 139 patients in the fresh frozen plasma group and 57 patients in the albumin group. Both the fresh frozen plasma and albumin groups received the standard therapy of the burn center, which was based on a standard protocol. Then, these patients were compared according to their clinical findings and mortality.

Results: There were no significant differences between the groups with respect to age, gender, and comorbidities. The laboratory findings, blood, urine, and wound culture results were also similar between groups. The mortality rate was higher in the group receiving albumin than in the group receiving fresh frozen plasma (78.9% and 33.8%, respectively; p=0.0007).

Conclusions: According to this study, there was a significant relationship between the improvement in mortality and the mode of colloid treatment in patients with major burns.

## Introduction

There is oedema formation that localises at the affected site in all trauma cases with burns, and when the burned total body surface area (TBSA) exceeds 30%, the fluid response becomes systemic [1]. In major burn patients, considerable amounts of fluid and plasma loss from the burned skin, which is proportional to the extensiveness and the shift of intravascular fluid to extravascular spaces in the first 24 hours after injury, results in a severe hypovolemic shock [1]. Prompt and thorough fluid resuscitation is required to prevent detrimental consequences [1].

There is almost a consensus on the delivery of Lactated Ringer’s solution for the fluid resuscitation of these patients. However, resuscitation with only crystalloids requires considerably large volumes, and larger volumes not only aggravate the generalised non-burn oedema but also cause longer post-burn intravascular plasma volume deficiency time [2]. This necessitates the addition of colloid treatment to burn shock management.

Colloid treatment elevates intravascular oncotic pressure, improves organ and tissue perfusion, especially for continuing renal perfusion, and acts as a volume expander for the intravascular space [3]. The main aim of adding colloid treatment to crystalloid treatment for major burn patients is to increase osmolarity. In turn, this aims to decrease crystalloid extravasation, reduce the volume of resuscitative crystalloids, and prevent over-resuscitation, which leads to compartment syndrome [4]. Although there is no consensus on the type, amount, dosing, and timing of the colloid treatment, the most widely accepted approach is to wait to deliver colloids until after the first 24 hours of injury [2].

Fresh frozen plasma (FFP) and albumin are two colloid treatment methods. Lawrence et al. reported that the addition of colloid treatment in the form of 5% human albumin to the Parkland formula decreased hourly fluid requirements and restored normal resuscitation ratios [5]. O’Mara et al. suggested that a combined crystalloid and colloid regimen as a form of FFP in the first 48 hours for large burn patients decreased fluid requirements and caused lesser increases in hyphen intraabdominal pressure [6].

However, there are conflicting data about the effect of colloids on the mortality of these patients and which form of colloid regimen is superior in the treatment of major burn patients in this regard. Therefore, we aimed to evaluate and compare the effects of albumin and FFP on treatment outcomes in our major burn patients.

## Materials and methods

This retrospective case-control study was conducted at Numune Training and Research Hospital, a tertiary centre in Ankara, Turkey. The Institutional Review Board of the hospital approved the study, and the universal principles of the Helsinki Declaration were applied. Two hundred and nine patients who had a burned TBSA of 30% or more during the periods of 2006-2007 or 2011-2012 were included in the study. At our institution, all patients were receiving albumin replacement as a volume expander in patients with burns over 30% of TBSA until 2008 as a part of hospital protocol. From 2008-2010, some patients were treated with albumin and some with FFP according to the respective surgeons’ preferences for burn-shock resuscitation. After 2010, FFP replacement as a colloid treatment was initiated as a part of the standard protocol for major burn patients in our hospital. This allowed us to define the two groups as an albumin-only resuscitation group (2006-2007) and an FFP-only resuscitation group (2011-2012).

None of the patients received colloids on the initial day of injury, which was in accordance with clinical protocol, and 13 patients who died within the first 24 hours of admission were excluded from the study. Demographic data, physical findings, medical histories, and laboratory results were obtained from hospital charts. Patients were grouped as receiving either albumin or FFP regarding their colloid treatment.

The descriptive statistics were shown as mean ± standard deviation for the continuous variables and as the quantity of subjects and their percentages (%) for the categorical variables. Whether the distribution of the variables was close to normal was explored using the Kolmogorov-Smirnov test and Levene’s test. Categorical variables were analysed with Pearson’s chi-square test. The significance of the difference between the groups in terms of averages was assessed using the T-test, and the significance of the difference in terms of median values was evaluated using the Mann-Whitney-U test. The Cox proportional hazards model method, the Log-Rank test, and the Kaplan-Meier method were used for survey analysis. The results were considered statistically significant when p<0.05 was used.

## Results

In the study period, there were 209 patients with TBSA greater than 30%. Thirteen patients died within the first 24 hours of injury without having colloid replacement and were not included in the study, leaving 196 patients to be included in the study. Of the whole study group, 24 (12.2%) were female and 172 (87.8%) were male. The mean age of the group was 36 ± 17.6 years. The burn aetiology was flame in 72.4% of patients, electric in 19.3%, and other causes in 8.3%. The mean burned TBSA was 53.9%, and the mean length of patients’ hospital stays was 25 days. The mortality rate for the whole study group was 46.9%.

Patients in the study were divided into two groups based on whether they received FFP (n=139) or albumin (n=57). For colloid therapy applied to the major burn cases included in the study process, easy access to FFP also affected the number of patients between groups according to year. There was no significant difference between the groups with respect to age and gender (Table 1). There was also no significant difference among the groups regarding co-morbidity with diabetes mellitus (p=0.672). The groups were similar regarding the agents that caused the burns. And also, there was a significant difference between the groups according to the burned TBSA and length of hospital stay (LOS) (Table 1).

**Table 1:** Demographic characteristics of FFP and albumin groups. FFP: fresh frozen Plasma; DM: diabetes mellitus; TBSA: total body surface area; LOS: length of hospital stay.

Variables	FFP group(n=139)	Albumin group(n=57)	P-value
Age (years)	35.6±17.0	38.2±19.3	0.382
Gender (n, %)			0.99
Female	17 (12.2%)	7 (12.3%)	
Male	122 (87.8%)	50 (84.7%)	
Burn agent (n, %)			0.074
Flame	93(66.9%)	49(85.9%)	
Electric	32(23.0%)	6(10.5%)	
Patients with DM	6(4.3%)	4(7%)	0.672
Others	14(10.0%)	2(3.5%)	
Burned TBSA (%)	52.0±17.3	58.7±20.0	0.020
LOS (days)	27.3±2.1	19.6±2.6	0.040

There were no significant differences between the groups regarding routine laboratory findings in the first evaluation of the patients. Both groups were also similar according to growth in the blood, urine, and wound sample cultures (Table 2).

**Table 2:** Laboratory characteristics of FFP and albumin groups. FFP: fresh frozen plasma; WBC: white blood cell; AST: aspartate aminotransferase, ALT: alanine aminotransferase; LDH: lactate dehydrogenase.

Variables	FFP group (n=139)	Albumin group (n=57)	P-value
Haemoglobin (g/dL)	14.3±3.3	15.0±3.8	0.208
Haematocrit (%)	42.3±9.6	44.1±11.1	0.247
WBC × 10^3^ mL	18.8±13.4	20.6±10.3	0.303
Platelets × 10^3^ mL	274±15.6	287±66.0	0.795
Albumin (g/dL)			
First value	3.1±9.4	2.8±10.3	0.062
Last value	2.3±1.1	2.1±1.8	0.146
Glucose (mg/dL)	135.6±56.2	141.2±44.9	0.472
AST (U/L)	109.2±26.3	89.9±16.0	0.613
ALT (U/L)	101.6±41.1	43.6±19.5	0.300
Total Bilirubin (µmol/l)	1.0±0.25	0.73±0.1	0.363
LDH	579.9±55.7	615.6±110	0.751
Growth in blood culture (n,%)	74(53.2%)	37(64.9%)	0.229
Growth in urine culture (n,%)	74(53.2%)	38(66.6%)	0.112
Growth in wound culture (n,%)	73(52.5%)	39(68.4%)	0.060

When patients were compared in terms of mortality, which was the main aim of this study, the albumin-receiving group had a significantly higher mortality rate than the FFP-receiving group (33.8% vs. 78.9%; p = 0.0007, respectively) (Table 3).

**Table 3:** The mortality rates of the FFP and albumin groups.

Variables	FFP group (n=139)	Albumin group (n=57)	P-value
Mortality rate (n,%)	47 (33.8%)	45 (78.9%)	0.0007

**Figure 1:**
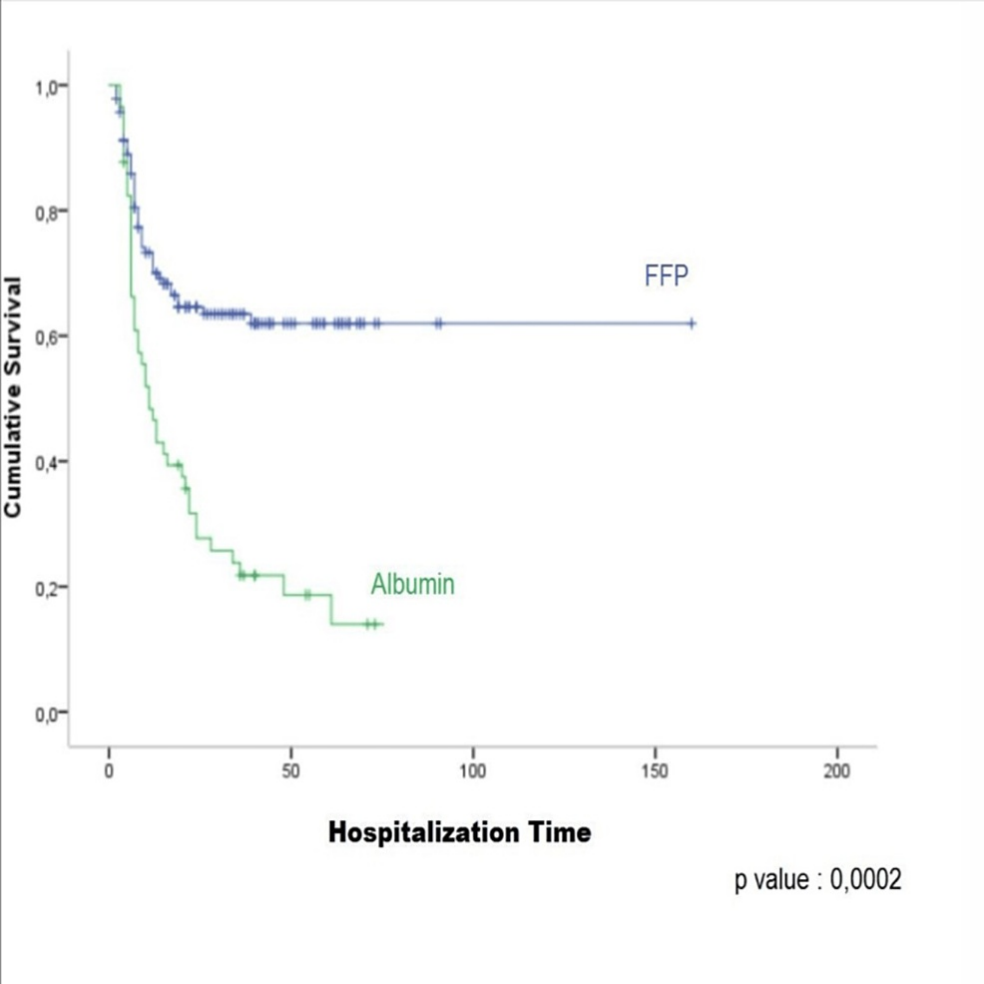
Kaplan-Meier survey analysis.

The mortality difference between the patients receiving albumin and FFP was analysed with the Cox proportional hazards model method and Kaplan-Meier survey analysis, and it was statistically significant in both survey tests (Figure 1). According to the Cox Proportional Hazard models, receiving FFP or albumin as colloid treatment, age, last albumin level, and burned TBSA were found to be independent risk factors for mortality (Table 4).

**Table 4:** Independent risk factors for the mortality with Cox regression method. TBSA: total body surface area.

	Adjust HR	%95 Confidence Interval		P-value
Colloid type	2.59	3.95	1.69	0.0001
Age	1.01	1.02	1.00	0.046
Albumin (first value)	0.98	1.00	0.96	0.15
Albumin (last value)	0.90	0.92	0.87	0.0002
Burned TBSA	1.02	1.03	1.01	0.0004

## Discussion

In the present study, we showed that mortality was significantly lower in the patient group that was treated with FFP and colloid than in the group that was given albumin, and in multivariate analyses, this effect was found to be independent of other risk factors. To the best of our knowledge and according to scientific databases, this is the first study that compares FFP and albumin treatments in major burn patients in terms of mortality.

Since April 2001, the patients hospitalised in our clinic have been given crystalloid therapy in the first 24 hours, and treatment with colloid has been added after the first 24 hours in accordance with the generally used protocols. In light of some in-vitro and laboratory animal studies published in the literature in recent years as well as clinical observations and experiences, the use of FFP instead of albumin is recommended for treatment with colloid in patients with large burns [3,7].

Trauma from burns affects mostly children and young adults. The most widely seen burn type in children under eight years of age is scalds due to hot liquid spills [8]. The most frequent causes of burns seen in children over eight years of age and adults are home fires, the use of inflammable fluids as igniters contrary to their purpose, and burns associated with smoking or alcohol [7]. The most common causes of workplace burns are chemicals or hot liquids, electrical burns, and melted or hot metals [8]. Adults were included in our study, which had a mean age of 36, and the most commonly seen cause of burns was flame.

It has been reported that mortality rates for major burn injuries have decreased in recent years due to a better understanding of burn pathophysiology and advanced monitoring techniques. While in the past, a 50% mortality rate was reported for a TBSA of 30% in healthy young adults, this went up to a TBSA of 80% today [9,10]. This fact indicates a great development in the management of patients with major burns compared to past years. However, mortality rates for major burns are still high in many countries across the world. While mortality has been reported at 46% in a study made in Australia, this rate has been reported at 54% in Italy for burns with a mean TBSA of 54.5±18.1% [9,10]. Looking at the patients as a whole in our study, the TBSA was 53.9% and the mortality rate was 46.9%, which is similar to the literature.

When the patients who received FFP and albumin as their treatment with colloid were divided into two groups, there were no demographic differences between the groups. The amount of fluid therapy practised in our clinic was primarily determined in view of the monitoring of urine discharge and then the monitoring of the central venous pressure, pulse, and blood pressure, as well as laboratory results and clinical evaluation. Previous studies have confirmed that the two most important factors leading to mortality after the first 24 hours are sepsis and multi-organ failure [10]. When the growth in blood, urine, and wound sites was compared in the groups receiving albumin and FFP in our study, no difference was found. The homogeneous distribution of the two groups was important in eliminating any difference that would affect mortality.

Albumin is the most important reason for oncotic pressure, which creates plasma colloid pressure. It is used in many intensive care procedures to reduce fluid leakage by increasing colloid pressure [11]. When albumin is used as a treatment with colloid, fluid volume retention develops in the whole body [11]. Consequently, pulmonary edoema and lung function disorders may occur [11]. Treatment with albumin has been shown to impair kidney functions. The possible mechanisms of such dysfunction include the impairment of glomerular filtration by the macroaggregates of albumin and decreased sodium and water excretion due to increased oncotic pressure in peritubular vessels caused by albumin [11]. Rashes, fever, and eruptions may occur as a result of the albumin treatment’s effect on the immune system. The albumin treatment has also been shown to decrease the immunoglobulin concentration in the blood [11]. Due to its disadvantages compared to other colloids in terms of effectiveness and cost, the use of albumin is gradually decreasing [12].

The initial and final albumin values of the patients in the albumin and FFP-receiving groups were similar in our study, which shows that the initial albumin value is not important in the preference of a colloid. The dropping albumin value after the burn shock makes the surgeons performing the resuscitation think that albumin should be replaced directly. However, the complications caused by albumin are well known [11,12]. As it is known that a large burn results in pulmonary complications in patients, we believe that albumin should not be used in this patient group because it may aggravate pulmonary edema, making the patient’s condition more critical [12]. The fact that there was no difference in the final blood albumin levels of the patients is an indication that neither of the two colloid resuscitations produced any difference in the final blood albumin levels of the patients. In other words, administering albumin or FFP does not affect the blood albumin level.

Thus, we concluded that using fresh frozen plasma to increase plasma colloid pressure was more advantageous in terms of mortality compared to the use of albumin, and this advantage was statistically significant for the prevention of mortality. The high levels of protein C and protein S found in fresh frozen plasma have been reported to have positive effects on surfactants in the lungs. In this way, protective action is produced on alveolar damage, and FFP will replace the coagulation factors [13]. This may also explain the lower mortality in the FFP group.

The hospitalisation time was longer for the patients who received fresh frozen plasma. We think that this was due to the fact that the lifespan of the patients receiving FFP therapy was prolonged. A graphical illustration of this is shown in the Kaplan Meier survey analysis. It is seen in that graph that patients receiving fresh frozen plasma live longer, and the hospitalisation time of patients receiving albumin therapy is shorter (Figure 1). Patients receiving albumin pass away at an early stage, making the average hospitalisation time shorter for this group. Therefore, it should be borne in mind that such short hospitalisation times are associated with death and not with successful recovery.

In multivariate analyses in our study, we found that the low final albumin value, age, and TBSA were independent factors for increasing mortality, which is similar to the literature. There are limited randomised studies with a small number of patients in the literature regarding whether albumin administration during the fluid resuscitation phase in burn patients has any effect on mortality. In a Cochrane meta-analysis, a significantly increased relative risk for mortality with albumin administration is found in subgroup analyses. However, it is demonstrated that albumin treatment did not show any benefit and had a neutral effect on mortality in burn patients in another recent meta-analysis [14,15].

Since the patient's capillary permeability increases, colloids are not considered appropriate in the first 24 hours. Albumin and FFP have been associated with lower volume requirements for initial resuscitation, lower intra-abdominal pressure, and a lower incidence of compartment syndrome; thus, these solutions may have a place in burn resuscitation, but additional evidence is needed to support their use. Multicenter randomized controlled trials of fluid resuscitation in major burns are still needed to define the best fluid therapy in this population.

The present study has several important limitations. Our study had a retrospective design and a low number of patients, and we compared the patients over different time periods that may affect the course of the disease.

## Conclusions

In conclusion, in this study, we compared the albumin and FFP treatments in major burn patients and demonstrated that although the final serum albumin level was similar between groups, the mortality rate was found to be significantly higher in the albumin group than the FFP group, and in multivariate analyses, this treatment modality effect was found to be an independent factor. To the best of our knowledge, this is the first study in the literature that compares FFP and albumin treatments in major burn patients in terms of mortality. However, further prospective randomised controlled studies with larger sample sizes are needed to assess the impact of treatment regimens on mortality in major burn patients.
